# Genetic variants in MUTYH are not associated with endometrial cancer risk

**DOI:** 10.1186/1897-4287-7-3

**Published:** 2009-01-26

**Authors:** Katie A Ashton, Anthony Proietto, Geoffrey Otton, Ian Symonds, Rodney J Scott

**Affiliations:** 1 Discipline of Medical Genetics, School of Biomedical Sciences, Faculty of Health, University of Newcastle, Australia and the Hunter Medical Research Institute, NSW, 2308, Australia; 2 Hunter Centre for Gynaecological Cancer, John Hunter Hospital, Newcastle, NSW, 2305, Australia; 3 School of Medicine and Public Health, Faculty of Health, University of Newcastle, NSW, 2305, Australia; 4 Division of Genetics, Hunter Area Pathology Service, John Hunter Hospital, Newcastle, NSW, 2305, Australia

## Abstract

Hereditary non-polyposis colorectal cancer (HNPCC), also known as Lynch syndrome, is an autosomal dominant inherited predisposition to a number of epithelial cancers, most notably colorectal and endometrial cancer. Outside of the context of Lynch syndrome there is little evidence for an autosomal dominant or recessive condition that predisposes to endometrial cancer. Recently, genetic variants in MUTYH have been associated with a recessive form of colorectal cancer, known as MUTYH associated polyposis or MAP. MUTYH is involved in base excision repair of DNA lesions and as such a breakdown in the fidelity of this process would necessarily not be predicted to result in a specific disease. At present there is little information about the role of MUTYH in other types of cancer and only one report indicating a possible relationship with endometrial cancer.

Similar to a previous study, we investigated a series of endometrial cancer patients to determine if MUTYH variants were over-represented compared to a series of healthy control subjects and to assess whether or not endometrial cancer risk could be explained by an autosomal recessive model of inheritance.

Two MUTYH mutations, Y165C and G382D, and three common MUTYH polymorphisms, V22M, Q324H and S501F, were genotyped in 213 endometrial cancer patients and 226 controls from Australia using real time PCR. Differences in genotype frequencies were compared using Chi-squared analysis and by calculating odds ratios and 95% confidence intervals.

Three endometrial cancer patients were identified with heterozygous MUTYH mutations (two G382D and one Y165C). No bi-allelic mutation carriers were identified. Two of the three patients' clinical characteristics were similar to those commonly identified in HNPCC and lend support to the notion that MUTYH mutations increase the risk of developing HNPCC related diseases. There was no difference in the five genotype frequencies of the endometrial cancer patients compared to the controls. The results of our study suggest that MUTYH is unlikely to be involved in the genetic basis of endometrial cancer but a possible association of MUTYH variants with HNPCC related diseases cannot be excluded.

## Background

MUTYH (MYH) is a DNA glycosylase which plays an essential role in the base excision repair (BER) pathway to prevent the accumulation of mutations that are a result of oxidative DNA damage [1]. In 2002, two autosomal recessive inherited mutations in MUTYH, Y165C and G382D, were associated with adenomatous polyposis and colorectal cancer [2], and several additional studies have confirmed that bi-allelic mutation carriers have an increased risk of developing colorectal cancer [3-5]. These two mutations are reported to account for approximately 86% of all variations in the MUTYH gene that are identified in Caucasians [6].

Some studies have suggested that mono-allelic changes in MUTYH increase colorectal cancer risk however this remains to be confirmed [3,4]. Furthermore, association studies assessing mono-allelic changes in MUTYH in combination with DNA mismatch repair genes, have revealed a possible relationship, specifically between hMSH2 and hMSH6, and an increased risk of developing colorectal cancer although this remains to be definitively confirmed [7,8]. Mutations in MMR genes are associated with hereditary non-polyposis colorectal cancer (HNPCC) which is an autosomal dominant inherited predisposition to colorectal cancer, endometrial cancer and a number of other malignancies [9]. Results from previous studies point towards a role of MUTYH mutations in HNPCC and suggest that they may be involved in a larger spectrum of disease involving extra-colonic cancers [7,8].

The role of MUTYH mutations in extra-colonic cancers has previously been reviewed and there are indications that this gene is associated with a broader spectrum of disease [6]. The report by Barnetson et al. (2005) focused on determining whether or not variants in MUTYH were related to endometrial cancer risk [6]. They identified one patient that was a compound heterozygote for Y165C and G382D. This patient had a sebaceous carcinoma which is a feature of Muir-Torre syndrome and is associated with MMR gene mutations. Five patients heterozygous for either Y165C or G382D were also identified. These MUTYH heterozygous mutation carriers did not harbour other pathogenic mutations, only a number of intronic variants. Since their conclusion that bi-allelic changes may increase susceptibility to endometrial cancer is based on one patient, these results need to be confirmed in a larger number of endometrial cancer cases.

In addition to the common MUTYH mutations, Y165C and G382D, three common polymorphisms in the Caucasian population have been identified: V22M, Q324H and S501F [2]. These polymorphisms have been suggested as being associated with an increased risk of developing colorectal cancer, however, it remains to be determined if these changes are tissue specific with respect to disease risk. Notwithstanding, these five MUTYH variants represent a significant proportion of the genetic variation present in MUTYH and warrant further investigation.

To confirm if the two common MUTYH mutations, Y165C and G382D and three common polymorphisms, Q324H, V22M and S501F are associated with endometrial cancer, 191 endometrial cancer patients were genotyped for these 5 variants.

## Materials and methods

### Study population

This study initially consisted of 213 consecutively recruited women with histologically confirmed endometrial cancer who presented for treatment at the Hunter Centre for Gynaecological Cancer, John Hunter Hospital, Newcastle, New South Wales, Australia between the years 1992 and 2005. Women that had additionally been diagnosed with breast cancer were excluded from this study.

The final analysis included 191 endometrial cancer patients. Data on reproductive and environmental risk factors including ethnicity, body mass index (BMI), diabetes, high blood pressure (HBP), age of diagnosis of endometrial cancer, age of menarche, age of menopause, other personal cancer history, family cancer history (Family history of cancer was defined as cancer in the index patient plus one or more 1st or 2nd degree relatives diagnosed with cancer), parity, breastfeeding, oral contraceptive use, chemotherapy, radiotherapy, hormone replacement therapy (HRT), smoking and alcohol use was collected using self reported questionnaires. Information regarding recurrence, stage, grade and histology of endometrial cancer was collected from the medical records. Two healthy anonymous control populations were used in this study from the Hunter Area of Newcastle. DNA samples were collected between the years of 1993 and 1997. For the Q324H, V22M and S501F polymorphisms, 226 patients were genotyped, and for the Y165C and G382D variants, 120 patients were genotyped and had a mean age of 51 years.

All participants provided informed written consent prior to participation in this study. Ethics approval was obtained from the Human Research Ethics Committee, University of Newcastle and the Hunter Area Research Ethics Committee, Hunter New England Health Service, Newcastle, New South Wales, Australia.

### DNA isolation

Genomic DNA was extracted from 10 ml EDTA blood as previously described [10].

### Molecular analysis

Genotyping of the five MUTYH polymorphisms Y165C, G382D, S501F, Q324H and V22M were performed on an ABI PRISM^® ^7500 Real-Time PCR System (PE Applied Biosystems, Foster City, CA), using primers and probes from Assay-by-Demand (Q324H and V22M) (assay ID: Q324H – C___27504565_10 and V22M – C___25955644_10) and Assay-by-Design (Y165C, G382D and S501F) (Applied Biosystems). The primers and probes for Y165C, G382D and S501F are listed in table 1. All assays were performed under universal conditions previously described [11]. Briefly, the assay functioned under universal conditions with each reaction containing: 50 ng DNA, 0.125 μl 40× Assay Mix and 2.5 μl TaqMan^® ^Universal PCR master mix made up to 5 μl with sterile water. The thermal cycling conditions were 50°C for 2 min, 95°C for 10 min, and 50 cycles of 92°C for 15 sec and 60°C for 1 min. Post PCR, the plate was scanned to allow discrimination between the different genotypes. The genotyping results were confirmed by a second laboratory research assistant and 5% of the samples were re-genotyped with 100% concordance. Any sample where a genotype could not be accurately assessed was re-genotyped. If it failed a second time, it was discarded from the analysis. The overall call rates were in the range from 96.0–100%.

**Table 1:** Real-Time PCR Assay-by-Design Primers and Probes for MUTYH Y165C, G382D and S501F

**SNP**	**Forward**	**Reverse**	**Wild Type Probe**	**Mutant Probe**
MUTYH S501 (C>T)	CAGCCTTCCAAAAGGTTCCCA	GCTGTGTGCATCAGTGGAGAT	VIC-CACGGAGAGGACAC	FAM-CACGGAAAGGACAC
MUTYH Y165C (A>G)	CCACAGGAAGGTGAATCAACTCT	CCTTACCTTCCGAGCTCCCT	VIC-CTGGGCTACTATTCT	FAM-GGGCTGCTATTCT
MUTYH G382D (G>A)	GACCCCTGCCTGGCT	GACGGGAACTCCCACAGT	VIC-CCTCTCAGGTCTGCTG	FAM-CCTCTCAGATCTGCTG

Note: The mutation/polymorphism is underlined in the probe sequences.

* The MUTYH S501F polymorphism is designed on the reverse strand.

### Statistical analysis

To determine differences in genotype frequencies between the cases and controls, chi-squared (χ^2^) statistics and odds ratios and 95% CIs were calculated. T-tests were used to determine differences in the age of diagnosis of endometrial cancer by genotype. The significance levels of all tests were set at p < 0.05 and were two-sided. All statistical analysis was performed with Intercooled STATA 8.2 (Stata Corp., College Station, TX, USA), SPSS Version 15 (SPSS Inc. Chicago, IL, USA).

## Results

The genotype frequencies were compared between the cases and controls for the two MUTYH mutations and the three MUTYH polymorphisms however no significant differences were observed (see table 2). We included endometrial cancer cases that were likely to be a result of tamoxifen treatment as they had previously been diagnosed with breast cancer. These patients did not alter the genotype frequency results for the three polymorphisms nor did these patients have a mutation in Y165C or G382D.

**Table 2:** The five MUTYH variants and their association with endometrial cancer risk

**Polymorphisms**	**Genotype**	**Cases n (%)**	**Controls n (%)**	**X^2^**	**OR (95% CI) and p value**
MUTYH Y165C (A>G)	AA	190 (99.5)	120 (100)		1.00 (reference)
	AG	1 90.5)	0 (0.0)	P = 0.43	0.53 (0.02–13.1) p = 0.43
	GG	0 (0.0)	9 (0.0)		
					
MUTYH G382D (G>A)	GG	189 (99.0)	118 (98.3)		1.00 (reference)
	GA	2 (1.0)	2 (1.7)	p = 0.6	41.6 (0.22–11.5) p = 0.63
	AA	0 (0.0)	0 (0.0)		
					
MUTYH V22M (G>A)	GG	172 (90.1)	194 (85.8)		1.00 (reference)
	GA	17 (8.9)	31 (13.7)		1.62 (0.86–3.02) p = 0.17
	AA	2 (1.0)	1 (0.4)		0.44 (0.04–4.94) p = 0.92
					
MUTYH Q324H (G>C)	GG	109 (57.1)	129 (59.2)		1.00 (reference)
	GC	71 (37.2)	75 (34.4)	p = 0.83	0.89 (0.59–1.35) p = 0.66
	CC	11 (5.8)	14 (6.4)		1.08 (0.4&-2.47) p = 0.86
					
MUTYH S501F (C>T)	CC	187 (97.9)	210 (96.8)		1.00 (reference)
	CT	4 (2.1)	7 (3.2)	p = 0.48	1.56 (0.45–5.41) p = 0.69
	TT	0 (0.0)	0 (0.0)		

For the pathogenic MUTYH mutations, Y165C and G382D, there were only 3 patients identified with heterozygous changes, 2 for G382D and 1 for Y165C. No bi-allelic changes were identified. Additionally, these women did not harbour any of the three common polymorphisms, V22M, Q324H or S501F. The characteristics of the MUTYH Y165C and G382D heterozygous mutation carriers are in table 3. Two of these patients had family histories of cancer that are possibly associated with a diagnosis of Lynch Syndrome as they both had first degree relatives with colorectal cancer. The other patient did not have any family history of disease.

**Table 3:** Characteristics of MUTYH Y165C and G382D heterozygous mutation carriers

**Characteristics**	**Y165C – patient 1**	**G382D – patient 2**	**G382D – patient 3**
Year of Birth	1933	1935	1951
BMI (kg/m^2^)	>30	25–30	25–30
Age of Diagnosis of Endometrial cancer (years)	71	70	52
Age of Menarche	15	16	15
Age of Menopause	55	45	52
No. of Children	0	3	2
Oral Contraceptive	Never Use	Never	Never
Other Diseases	High Blood Pressure Ovarian Cancer Colorectal Cancer	High Blood Pressure Diabetes	Diabetes
Family History of Sister Cancer	Colorectal cancer Mother & Maternal Aunt Breast Cancer	Father Colorectal Brother Leukaemia	None
Stage of Cancer	Unknown	1B	Mixed Mullerian Malignant Tumour
Grade of Cancer	1	1	3
Histology	Adenocarcinoma	Adenocarcinoma	Mixed Mullerian Malignant Tumour
Recurrence	None	None	None
Smoker	Never	Current	Never
Alcohol	Non-Drinker	Non-Drinker	Non-Drinker

T-tests were used to evaluate the influence of the five MUTYH polymorphisms on the age of diagnosis of endometrial cancer. No significant differences were observed (data not shown).

## Discussion

Currently, patients with multiple colorectal adenomas, no APC gene mutation or HNPCC related colorectal cancer and no MMR gene mutation are recommended to undergo testing for germline mutations in MUTYH. It is not known whether mutations or polymorphisms in MUTYH are specific for colorectal cancer or if they encompass a larger spectrum of disease, especially that which is over-represented in Lynch Syndrome. Since the MMR genes, hMSH2 and hMSH6 are associated with HNPCC and evidence suggests that they directly interact with MUTYH, it is possible that MUTYH mutations are related to HNPCC extra-colonic cancers, specifically endometrial cancer.

This study identified three endometrial cancer patients with heterozygous Y165C or G382D changes in MUTYH. No bi-allelic mutation carriers were identified in 191 endometrial cancer patients, however the monoallelic mutation carriers could possibly have other rare alterations that were not investigated. Our results are similar to a previous study which found one bi-allelic mutation carrier and five patients with heterozygous changes [6]. Two of the three patients with heterozygous changes had a family history of colorectal and endometrial cancer which could possibly reflect their relationship with HNPCC.

We analysed three additional common polymorphisms in MUTYH, Q324H, V22M and S501F, but did not find a second variant in the women with heterozygous Y165C or G382D mutations. It is highly unlikely that these women harbour another as yet unidentified polymorphism since the analysis of these five polymorphisms accounts for a large majority of genetic variation in MUTYH in Caucasians. Additionally, we compared the genotype frequencies for all five MUTYH variants but did not find a significant difference between the endometrial cancer group and the controls which suggests that these variants do not appear to increase the risk of developing endometrial cancer, although a larger population is required to confirm this statement.

Our results suggest that the MUTYH mutations, Y165C and G382D, only account, if at all, for a minority of endometrial cancer cases. We can not rule out the possibility that these mutations may act as modifiers of disease penetrance since they both have been predicted to interact functionally with hMSH2 and hMSH6. Furthermore, it is not clear whether there are tissue specific differences in disease expression that may be related to environmental influences that are specific for each anatomical site. Recently, a study of over 600 breast cancer cases also revealed similar results to our own in that no bi-allelic mutations were identified and there was no change in the risk of disease in mono-allelic carriers. Together, the combined results suggest that MUTYH is not associated with an increased risk of breast cancer [12] or endometrial cancer.

In conclusion, our results in combination with Barnetson et al. (2005) [6] reveal that variation in MUTYH is very limited in endometrial cancer and does not appear to alter the susceptibility to sporadic endometrial cancer however MUTYH variants possibly have some role in HNPCC related disease.

## Competing interests

The authors declare that they have no competing interests.

## Authors' contributions

KAA carried out the molecular genetic studies and wrote the first drafts of the manuscript. AP, GO and IS contributed to the collection and clinical histories of the patients. RJS conceived the study and participated in its design and coordination. All authors have read and approved the final version of the manuscript.
